# The Influence of Mid-Event Deception on Psychophysiological Status and Pacing Can Persist across Consecutive Disciplines and Enhance Self-paced Multi-modal Endurance Performance

**DOI:** 10.3389/fphys.2017.00006

**Published:** 2017-01-24

**Authors:** Daniel Taylor, Mark F. Smith

**Affiliations:** School of Sport and Exercise Science, University of LincolnLincoln, UK

**Keywords:** deception, triathlon, multisport, pacing, rating of perceived exertion, affect, rating of perceived effort, teleoanticipation

## Abstract

**Purpose:** To examine the effects of deceptively aggressive bike pacing on performance, pacing, and associated physiological and perceptual responses during simulated sprint-distance triathlon.

**Methods:** Ten non-elite, competitive male triathletes completed three simulated sprint-distance triathlons (0.75 km swim, 500 kJ bike, 5 km run), the first of which established personal best “baseline” performance (BL). During the remaining two trials athletes maintained a cycling power output 5% greater than BL, before completing the run as quickly as possible. However, participants were informed of this aggressive cycling strategy before and during only one of the two trials (HON). Prior to the alternate trial (DEC), participants were misinformed that cycling power output would equal that of BL, with on-screen feedback manipulated to reinforce this deception.

**Results:** Compared to BL, a significantly faster run performance was observed following DEC cycling (*p* < 0.05) but not following HON cycling (1348 ± 140 vs. 1333 ± 129 s and 1350 ± 135 s, for BL, DEC, and HON, respectively). As such, magnitude-based inferences suggest HON running was more *likely* to be slower, than faster, compared to BL, and that DEC running was *probably* faster than both BL and HON. Despite a trend for overall triathlon performance to be quicker during DEC (4339 ± 395 s) compared to HON (4356 ± 384 s), the only significant and *almost certainly* meaningful differences were between each of these trials and BL (4465 ± 420 s; *p* < 0.05). Generally, physiological and perceptual strain increased with higher cycling intensities, with little, if any, substantial difference in physiological and perceptual response during each triathlon run.

**Conclusions:** The present study is the first to show that mid-event pace deception can have a practically meaningful effect on multi-modal endurance performance, though the relative importance of different psychophysiological and emotional responses remains unclear. Whilst our findings support the view that some form of anticipatory “template” may be used by athletes to interpret levels of psychophysiological and emotional strain, and regulate exercise intensity accordingly, they would also suggest that individual constructs such as RPE and affect may be more loosely tied with pacing than previously suggested.

## Introduction

During sprint-distance triathlon, an athlete's overall finishing time comprises a 0.75 km swim, 20 km cycle, and 5 km run, each of which is separated by only a brief period of “transition”. Each discipline imposes unique residual demands on the next (Peeling and Landers, 2009) and differs in its contribution to total time (swim ~17%, cycle ~51%, run ~27%; Taylor et al., 2012; Taylor and Smith, 2013). An optimum pacing strategy during triathlon therefore needs to balance the relative intensity within each discipline against the benefits or consequences of these intensities in relation to overall finishing time and/or position (Edwards and Polman, 2013). Indeed, completing the swim at the highest sustainable pace (i.e., 100% of isolated time-trial pace) has been shown to significantly impair overall short-distance triathlon performance time (~1 min 45 s), compared to swimming at 80–85% of isolated time-trial intensity (Peeling et al., 2005). Thus, it would seem that maintaining a reserve capacity throughout the swim is essential if overall triathlon performance is to be optimized. Conversely, Suriano and Bishop (2010) have demonstrated that aggressive pacing of the cycle section (i.e., equivalent to mean power output during an isolated time-trial) significantly impairs subsequent running speed but enhances total cycle-run time over the sprint-distance format. Although this study failed to include an initial swimming leg, the findings appear to support the view that cycling at the highest sustainable intensity may be the best strategy to optimize overall performance during short-distance triathlon events.

Despite these points, it is not yet clear how expectations, beliefs and perceptions might influence the pursuit, and success, of aggressive mid-race pacing strategies during multi-modal endurance performance. Indeed, it is not unreasonable to suggest that attenuations in performance following an aggressively paced cycling section may, at least partly, be the result of triathletes having preconceived expectations of this strategy and the need to reduce their subsequent (i.e., running) exercise intensity as a result (Hausswirth et al., 1999). As such, it is thought that athletes are likely to perceive a higher than usual mid-event pace, and associated levels of psychophysiological strain, as posing an increased threat to the successful completion of an exercise task and, therefore, as having a “price to pay” at a later stage of performance (i.e., reduction in subsequent pace to restore anticipated levels of psychophysiological strain, and so reduce risk of premature exhaustion or harm) (de Koning et al., 2011; Cohen et al., 2013; Micklewright et al., 2015). However, whether altering the perceived “riskiness” of aggressive pacing during cycling can help to ameliorate impairments in subsequent running, and thus enhance overall triathlon performance, is yet to be elucidated.

It has been suggested that practically meaningful changes in triathlon running may result from deceptive pace manipulation, equivalent to the smallest worthwhile change in performance (i.e., typical within-athlete variability or coefficient of variation) (Taylor and Smith, 2014). More specifically, Taylor and Smith (2014) have demonstrated that run performance during sprint-distance triathlon may be enhanced by the imposition of a deceptively aggressive starting strategy (3% faster than baseline performance), when compared to more conservative approaches to initial pace deception (3% slower than, and equal to, baseline performance). These findings would appear to support the view that individual's typically maintain some form of reserve capacity during self-paced exercise and perform at a relative intensity somewhat below their task-specific maximum capacity, even when their intention is to optimize performance (Stone et al., 2012; St Clair Gibson et al., 2013). Furthermore, this study adds weight to the idea that an individual's expectations, beliefs and perceptions play an important role in how much reserve capacity they are willing to utilize during self-paced multi-modal exercise tasks. Given these points, it is reasonable to suggest that deceptively aggressive bike pacing may allow triathletes to maximize their performance within this discipline, help to avoid the reductions in running performance which may typically follow this strategy (i.e., Suriano and Bishop, 2010) and, in turn, optimize overall event time. However, as far as we are aware there are no studies to date which have examined the effects of deceptively aggressive bike pacing on triathlon performance.

There is a similar lack of experimental evidence regarding the relative importance of different perceptual responses to pacing and performance during multi-modal exercise (Wu et al., 2014). Indeed, the aforementioned study of Taylor and Smith (2014) reported non-significant trends for increased ratings of perceptual strain during the first 1.66 km of triathlon running when deceptively higher speeds were imposed, and vice-versa. Beyond this point (i.e., during self-paced completion of the run), a common pattern of development for many perceptual responses was seen between deceptive run conditions. These observations provide tentative evidence of the robustness that different psychophysiological and emotional responses have to manipulations of expectations and beliefs, and offer an insight into the relative importance of these perceptions in contextualizing or “framing” past, present, and future demands (and pacing) during multi-modal exercise. However, it is apparent that the findings and conclusions of Taylor and Smith (2014) may have been limited by the timing of deceptive pace manipulation relative to the simulated triathlon overall (i.e., between 72–81% of total time), combined with the relative contribution of the run section to overall performance time in the event (i.e., ~28% of total time). As such, the scope for deceptive manipulations of pace to make a meaningful difference to triathlon performance and distinguish the relative importance of perceptual mediators to pace regulation and reserve maintenance may therefore be greater during the earlier swim and cycle sections of the event.

With the aforementioned points in mind, and given that the cycling section typically contributes the highest proportion of overall triathlon time, this study examined the effects of deceptively aggressive bike pacing on performance, physiological and perceptual responses, and pacing during simulated sprint-distance triathlon. More specifically, it was hypothesized that completing the cycling section closer to the highest sustainable intensity (i.e., mean isolated time trial power output) would improve previous best simulated triathlon performance, irrespective of whether triathletes were made aware of this pacing strategy or not. However, it was also hypothesized that making triathletes aware of this aggressive cycling strategy would impair subsequent run and overall performance, relative to a deceptive pacing condition.

## Methods

### Participants

Ten non-elite, trained male triathletes gave written, informed consent to participate in this study, with a mean (±*SD*) age, body mass, stature, and peak oxygen uptake ($\dot{\text{V}}$O_2peak_) of 36.8 ± 8.9 years, 1.79 ± 0.08 m, 76.3 ± 7.2 kg, and 54.3 ± 5.7 ml·kg^−1^·min^−1^, respectively. Participants had been competing in triathlons for a minimum of 12 months and were all in their “off-season” throughout the study. The training completed by the group during the study period averaged 1.4 h·wk^−1^ (3.2 km·wk^−1^) swimming, 2.3 h·wk^−1^ (84.0 km·wk^−1^) cycling, 2.2 h·wk^−1^ (21.7 km·wk^−1^) running, in addition to 1.3 h·wk^−1^ of strength and conditioning. Before the completion of any data collection, all participants completed a medical history questionnaire and, having had the research procedures, requirements, benefits, and risks explained to them, they each provided written, informed consent. At this initial stage participants were told, incorrectly, that the intention of the study was to establish the reliability and validity of simulated sprint-distance triathlon performance, and associated physiological and perceptual responses. All study procedures were approved by the institutional ethics committee and, in line with internationally recognized ethical standards for deceptive sport and exercise science research (Harriss and Atkinson, 2015), all participants were fully debriefed upon completion of all trials, informed how they were deceived and why such deception was necessary, and were given the option to withdraw their data. Participants were permitted to follow their usual training regime throughout the study but were instructed to avoid training in the 24 h preceding each trial. As such, participants were asked to record and manage their training and dietary/fluid intake in order to maintain a consistent approach to the 24 h period preceding each trial.

### Procedure and apparatus

Participant's completed a total of eight testing sessions each, with the first four consisting of an “all-out” (non-drafted) swimming time-trial performed in their usual (25 m) training pool, separate incremental running and cycling tests to volitional exhaustion to establish each participant's peak physiological (i.e., $\dot{\text{V}}$O_2peak_ and heart rate [HR_peak_]) and performance (i.e., running speed [V_max_] and power output [W_max_]) characteristics, and a “race pace” familiarization of the sprint-distance triathlon simulation (750 m swim, 500 kJ bike, 5 km run) that they would be required to complete during subsequent experimental triathlon trials. Having completed all preliminary testing, each participant then performed an isolated cycling time-trial (TT) which required the completion of 500 kJ of work as quickly as possible. In light of the work by Suriano and Bishop (2010), it was reasoned that including this trial would determine each participant's highest sustainable intensity during a 500 kJ time-trial and would therefore serve as a benchmark with which to interpret cycling performance (and associated physiological or perceptual responses) during subsequent simulated triathlon trials. The remaining trials required each participant to complete three separate simulated sprint-distance triathlons (0.75 km swim, 500 kJ bike, 5 km run). These were performed at the same time of day, separated by an average of 8 days (range, 3–14 days) and completed in a maximum of 21 days. During all laboratory trials, swimming was performed in a temperature-controlled flume (Fastlane, Endless Pools, UK; water temperature ~24.3°C), with all cycling and running completed in an adjacent environmentally controlled room (mean air temperature 21.7°C and mean relative humidity 56.5% across all trials). Electric fans were also placed ~1 m in front of participants to provide continuous and consistent levels of additional air ventilation (~4 m·s^−1^, CIMA AR-816 digital anemometer) throughout all cycling and running sections. Cycling was completed on an electromagnetically braked ergometer (SRM; Jülich, Welldorf, Germany) and running was performed on a motorized treadmill (HPCosmos, Traunstein, Germany).

The first simulated triathlon trial served to establish personal best “baseline” performance (BL). Swimming was completed at a fixed intensity equivalent to 90% of the average velocity recorded during each participant's preliminary “all-out” 750 m time-trial. As Peeling et al. (2005) have suggested that sprint-distance triathlon performance may be optimized by athletes maintaining this swimming intensity it was considered as a valid way to incorporate this discipline into short-distance triathlon simulations (Stevens et al., 2013). Having completed the swim and exited the flume participants were instructed to complete the remainder of the simulated triathlon (including transitions) as quickly as possible, as they would during competitive performance. The second and third simulated triathlon trials were completed in a randomized and counterbalanced order, with each requiring participants to maintain a prescribed power output for the entirety of the 500 kJ cycling section, before completing the run as quickly as possible. During both of these trials the (average) power output that participants were required to maintain was 5% greater than that achieved during BL performance. However, participants were only correctly informed of this prior to and during one of these trials (HON). Before (and during) the alternate trial (DEC), participants were misinformed that they would be required to maintain a power output equal to that of their BL performance. As such, the on-screen feedback provided during this trial was manipulated so that it displayed average and real-time power output values 5% lower than they truly were, as measured by the SRM ergometer. The only other feedback provided during each cycling performance was verbal confirmation of every 5% (25 kJ) of total work completed. It was reasoned that informing participants of the HON pacing manipulation at this stage of the study (rather than during the pre-study period) would have helped to facilitate their best-possible BL performance and avoid any “holding back,” in light of the greater demands that performing “as fast as possible” would likely lead to during subsequent trials (i.e., HON).

The magnitude of deception employed was selected based on the previously established coefficient of variation (CV) for power output during simulated triathlon cycling (CV = 4.8%; 95% CI = 3.4–8.4%) and associated estimates of sample size requirements (Taylor et al., 2012). As such, it was reasoned that a 5% manipulation of power output would allow for the imposition of a worthwhile performance change, whilst also being subtle enough to avoid any detection by participants across trials. Furthermore, the aggressiveness of this imposed pacing strategy (relative to TT performance) was comparable to previous non-deceptive manipulations of triathlete pacing during simulated sprint-distance cycle-run trials (Suriano and Bishop, 2010).

Throughout all running performances, the treadmill was interfaced with the computer-based NetAthlonTM software package (WebRacing Inc., Madison, WI) which was, in turn, projected onto a large monitor positioned in front of the treadmill. This provided a virtual representation of each participants progress over a flat 5 km run course in the form of an on-screen avatar (viewed from a second person perspective), in addition to numerical feedback regarding distance covered, current speed and average speed. In addition to this feedback, participants were informed prior to HON and DEC trials that they would be racing against a second on-screen avatar during the run which represented a replay of their BL performance. More specifically, participants were instructed to try their best to beat (or at least match) this on-screen “opponent.” The view seen by each participant was always of the avatar representing their current performance. This meant that they were only able to see both avatars if they were performing worse than their BL trial (i.e., in a “chase” position). With this in mind, the distance separating both avatars was constantly displayed on-screen so that participants were able to keep track of their relative performance and respond to any changes in pace that were made during the BL trial. Upon completion of each run, the NetAthlonTM software stored distance, speed and time data at 1 s intervals for subsequent analysis.

The duration of first and second transition during HON and DEC trials replicated those recorded during BL performance (221 ± 31 and 93 ± 22 s, respectively) and were comparable to previous studies of simulated triathlon performance (Hausswirth et al., 2010; McGawley et al., 2012; Taylor et al., 2012; Taylor and Smith, 2014). The methods adopted to examine the respiratory responses of participants (see Section Physiological responses) meant that fluid intake was only possible during the cycling section of simulated triathlon. As such, participants were allowed to consume water *ad libitum* whenever these measures were not being recorded. More specifically, participants were instructed to drink as dictated by their levels of thirst, which is suggested as a more important factor to control during triathlon simulations than specific measures of hydration status (Noakes, 2010; Stevens et al., 2013). In any case, there were no significant differences in the volume of water consumed by each participant during simulated triathlon (or isolated TT) performances (mean volume 317 ± 177 ml across trials; *p* > 0.05).

#### Physiological responses

During all laboratory trials, breath-by-breath measurements of oxygen uptake ($\dot{\text{V}}$O_2_), respiratory exchange ratio (RER) and ventilation ($\dot{\text{V}}$_E_) were obtained (Cortex Metalyzer, Leipzig, Germany), alongside heart rate (HR; RS_400_, Polar Electro Kempele, Finland) and fingertip capillary blood lactate concentration ([BLa^−^]; Lactate Pro 2, Arkray, Japan). Prior to each laboratory trial participants fitted a HR transmitter belt underneath their triathlon suit, with baseline measurements then obtained for [BLa^−^] and body mass. During simulated triathlon trials, measures of [BLa^−^] were obtained post-swim, at the end of every 100 kJ cycle section completed, and upon completion of each 1.66 km section of the run. These measures were also taken at the end of every 100 kJ during isolated TT performance. Body mass was measured immediately upon completion of each experimental trial. During isolated TT and simulated triathlon trials, the gas analysis system was fitted to participants immediately before they began cycling, by means of a leak-free face-mask and head-strap. However, to allow for fluid intake, this face-mask was removed from participants between 75–125, 175–225, 275–325, 375–425, and 475–500 kJ of the bike. During simulated triathlon trials this system (i.e., face-mask) was then reapplied at the end of second transition (i.e., once participants had mounted the treadmill) and was kept on for the duration of the run. Following each experimental trial, cardiorespiratory data was interpolated to 1 s averages using the manufacturer's software to match the frequency of this data with that of cycling power output and running speed. Mean HR values were determined for each triathlon discipline, whilst mean values for respiratory data were established for both the bike and run sections. In order to profile discipline-specific cardiorespiratory responses, data were averaged for 50–75 kJ of every 100 kJ cycle section completed and for each 1.66 km section of simulated triathlon running.

#### Perceptual responses

During each experimental trial, verbal ratings of perceived exertion, effort, muscular pain, breathlessness, thermal discomfort, affect, and arousal were obtained using the same scales and instructions as outlined by previous studies of sprint-distance triathlon (Taylor and Smith, 2013, 2014). Whilst the relative order of these scales remained the same throughout the study, the first scale presented in the sequence was randomized and counterbalanced for each participant, so as to minimize the interference between the relatively high number of separate perceptual responses. In the final 100 m of each triathlon swim, participants were prompted by an underwater visual signal to consider (and memorize) their perceptual status so that they could provide verbal responses to each scale during first transition. Perceptual responses were then obtained at the end of every 100 kJ cycle section and upon completion of each 1.66 km section of the run. These measures were also taken at the end of every 100 kJ during isolated cycling time-trials.

### Statistical analysis

All analyses were conducted using SPSS for Windows (Version 22, SPSS Inc., Chicago, USA) and Microsoft Excel (Microsoft Excel, 2007). A series of one-way repeated-measures ANOVA's were used to examine differences in swim, cycle, run and overall performance measures between BL, HON, and DEC triathlon trials, and to establish whether any performance differences existed between isolated cycling time-trials and the cycling section of each simulated triathlon. The same method of analysis was used to examine discipline-specific differences between trials in relation to the mean physiological and perceptual responses observed. In order to better consider the practical significance of results, data was also assessed by way of magnitude-based inferences (Batterham and Hopkins, 2006). Such analysis, performed using a published spreadsheet (Hopkins, 2003), provides quantitative (%) chances of “positive,” “trivial,” or “negative” effects between trials, based on the 90% confidence interval of the change value relative to a predetermined smallest worthwhile effect. With regards to cycling, running and overall performance data, the smallest worthwhile change values were based on those established by Taylor et al. (2012) during simulated sprint-distance triathlon performance of non-elite athletes (~2.4, ~0.6, and ~1.2%, respectively). Likewise, the smallest worthwhile changes in physiological responses established by Taylor et al. (2012) were used to make magnitude-based inferences regarding these measures. However, given their lack of established CV values during triathlon, the smallest worthwhile change for each perceptual measure was set relative to 0.2 times the pooled between-subject *SD* (Hopkins, 2000).

Two-way within-subjects (trial x distance) ANOVA's were used to establish main effects of cycling condition and distance completed using mean 100 kJ section values for power output, $\dot{\text{V}}$O_2_, $\dot{\text{V}}$_E_, RER, [BLa^−^], HR, perceived exertion, effort, muscular pain, breathlessness, affect, arousal, and thermal discomfort as dependent variables. The same analysis was used to examine data obtained during the running section of each simulated triathlon trial, using mean 1.66 km section values for speed and the same physiological and perceptual measures as dependent variables. Repeated measures ANOVA's were then used to identify changes in these variables during the course of each discipline. If the Mauchly test indicated a violation of sphericity then analysis of variance was adjusted using the Greenhouse–Geisser correction factor to reduce the likelihood of type I error. Where appropriate, Bonferroni-adjusted *post-hoc* tests were used to identify specific differences within and between trials. For all statistical procedures the level of significance was set at *p* < 0.05 and adjusted accordingly. All data are expressed as mean ± standard deviation and effect sizes for ANOVA outcomes as partial eta squared (η_*p*_^2^).

## Results

### Performance measures

As summarized in Table 1, there were no statistically significant differences in cycling time between HON, DEC, and TT, though each of these trials was significantly faster compared to BL (*p* < 0.05). As such, mean power output was significantly higher during TT, HON, and DEC, vs. BL (246 ± 34, 236 ± 33, and 236 ± 33, vs. 225 ± 32 W, respectively, *p* < 0.05). These power output values corresponded to 71, 65, 68, and 68% of W_peak_, for TT, BL, HON, and DEC, respectively. Mean running speed during each triathlon trial corresponded 77, 77, and 78% of V_peak_, for BL, HON, and DEC, respectively. Although these values suggest a trend for faster run performance during DEC, compared to both BL and HON, this was only statistically significant in comparison to BL (*p* < 0.05). Similarly, whilst there was a non-significant trend for overall triathlon time to be shorter during DEC than HON (by ~17 s), the only statistically significant differences were between each of these trials and BL, which was between 2 and 3% slower overall than both DEC and HON (*p* < 0.05).

**Table 1:** **Mean ± ***SD*** overall and discipline-specific performance times during each simulated triathlon and isolated time-trial (***n*** = 10)**.

	**Swim (s)**	**Cycling (s)**	**Run (s)**	**Overall (s)**
TT	–	2067 ± 312^b^	–	–
BL	848 ± 99	2270 ± 368^a^^,^ ^c^^,^ ^d^	1348 ± 140^c^	4465 ± 420^c^^,^^d^
DEC	848 ± 99	2158 ± 344^b^	1333 ± 129^b^	4339 ± 395^b^
HON	848 ± 99	2159 ± 343^b^	1350 ± 135	4356 ± 384^b^

*NB, Significantly different from; TT*,

a *p < 0.05; BL*,

b *p < 0.05; DEC*,

c *p < 0.05; HON*,

d *p < 0.05*.

**Figure 1:**
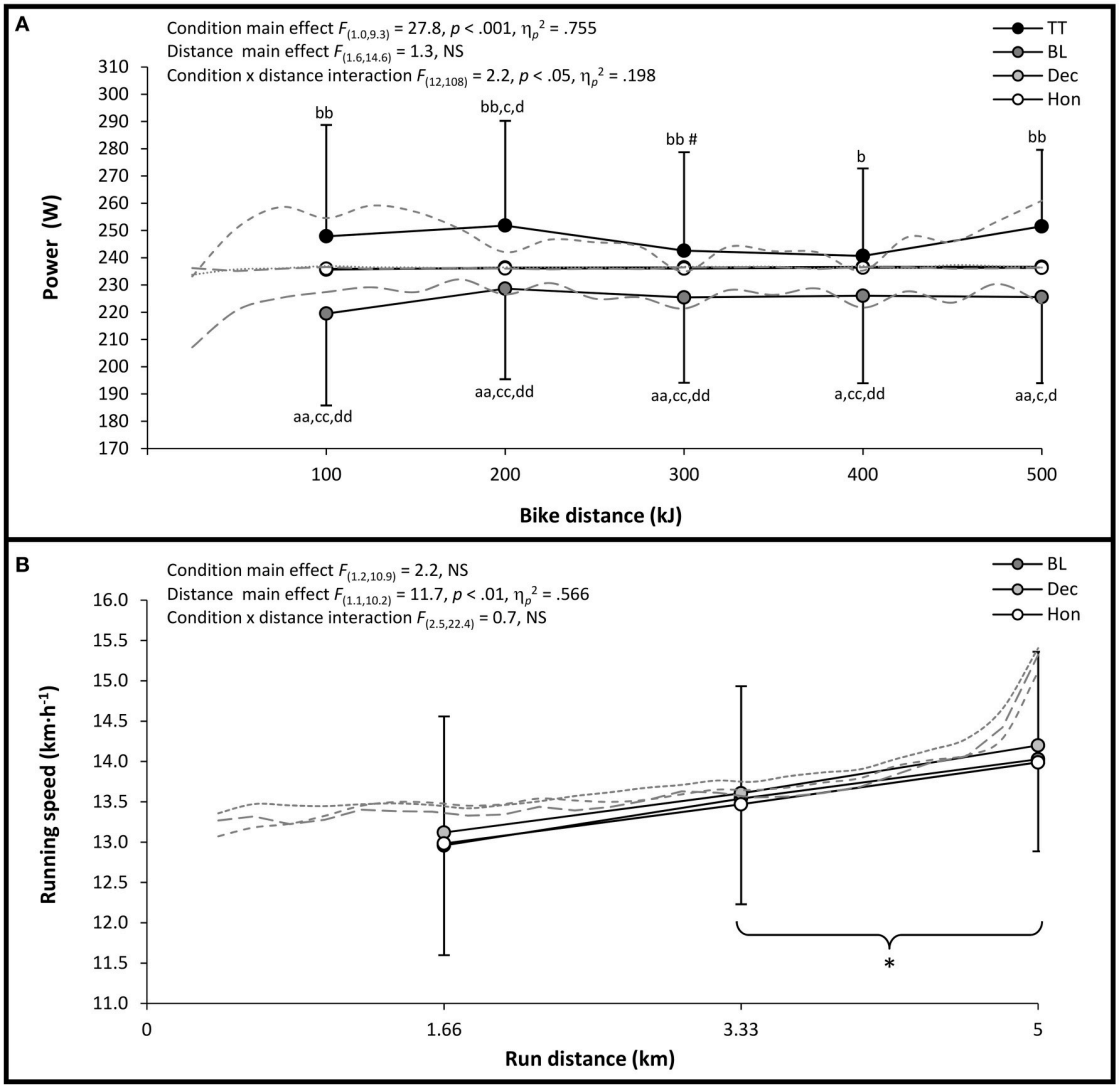
**(A)** Mean ± *SD* power output for each 100 kJ (solid lines) and 25 kJ (dashed lines) completed in each cycling condition, **(B)** Mean running speed for each 1.66 km (solid lines) and 200m (dashed lines) section completed in each triathlon trial. Significantly different from; TT, ^a^*p* < 0.05,^aa^*p* < 0.01; BL, ^b^*p* < 0.05, ^bb^*p* < 0.01; DEC, ^c^*p* < 0.05, ^cc^*p* < 0.01; HON, ^d^*p* < 0.05, ^dd^*p* < 0.01; initial value, ^*^p < 0.05; previous value, ^#^*p* < 0.05, (parentheses indicate significance in all conditions).

Repeated-measures ANOVA showed no main effect on power output for cycling distance, but did reveal a significant main effect for cycling condition and a significant condition × distance interaction, indicating differences across conditions in power output profiles when plotted against distance covered (Figure 1A). This assertion was supported by *post-hoc* analysis which highlighted a consistently higher power for each 100 kJ section during TT vs. BL. Although the pacing profiles during TT and BL developed in a similar (i.e., parallel) manner for much of the cycling bout, it was also evident that the marked increase in power output observed during the final 50 kJ of TT was absent during BL. During triathlon running, repeated-measures ANOVA revealed a significant main effect on speed for distance, but no main effect for condition and no condition × distance interaction (Figure 1B). As such, *post-hoc* analysis highlighted significant increases in speed for each successive 1.66 km section (*p* < 0.05) which culminated in an apparent “end-spurt” in the final 600 m of all triathlon trials.

With regard to the practical significance of performance differences, magnitude-based inferences suggest that cycling time and power output were *almost certainly* better during TT, DEC, and HON, in comparison to BL (i.e., 100% likelihood of each being meaningfully faster than BL). Whilst DEC and HON cycling performances were *probably* worse compared to that of TT (i.e., 90% likelihood), there were *almost certainly* no performance differences between the DEC and HON cycling (i.e., 100% likelihood). Interestingly, whilst any practically important difference appeared *unclear*, it was more likely that HON running performance was meaningfully slower, than faster, vs. BL (i.e., 28:57:15% likelihood of HON being practically slower, of trivial difference, or practically faster than BL). On the other hand, DEC running performance was *probably* faster than both BL and HON (i.e., 89 and 79% likelihood, respectively). In terms of overall triathlon performance, there was *almost certainly* no difference between DEC and HON (i.e., 100% likelihood), although both were *almost certainly* faster vs. BL (i.e., 100% likelihood of each being meaningfully faster than BL).

Further to these findings, post-experimental debriefing revealed that all participants; (i) failed to identify the aggressive manipulation of cycling power output during DEC and, similarly (ii) believed that cycling intensity was highest (i.e., “most difficult”) during their HON performance.

### Physiological measures

Table 2 summarizes the mean physiological responses during all triathlon and isolated cycling trials. There were no significant differences in mean physiological responses (i.e., HR and [BLa^−^]) elicited by the swim section of each simulated triathlon (*p* > 0.05). Mean cycling intensity during each trial corresponded to 91, 85, 87, and 87% of HR_peak_, and 87, 81, 83, and 82% of $\dot{\text{V}}$O_2peak_, for TT, BL, HON, and DEC, respectively. As such, comparisons of each cycling bout revealed that physiological responses during TT were significantly higher than those recorded during BL (*p* < 0.05). Furthermore, the greater demands of HON and DEC cycling were reflected in a number of elevated physiological responses compared to BL, particularly that of [BLa^−^].

**Table 2:** **Mean ± SD physiological responses during triathlon and TT trials (***n*** = 10)**.

	**$\dot{\text{V}}$O_2_ (L·min^−1^)**	**$\dot{\text{V}}$_E_ (L·min^−1^)**	**RER**	**HR (b·min^−1^)**	**[BLa^−^] (mmol·L^−1^)**
**SWIM**					
BL				115 ± 18	3.4 ± 2.0
DEC				113 ± 15	3.2 ± 1.5
HON				113 ± 16	3.2 ± 1.5
**CYCLE**					
TT	3.35 ± 0.40^b^^,^^c^	109.74 ± 22.38^b^	1.00 ± 0.04^b^	155 ± 11^b^^,^^c^^,^^d^	6.9 ± 3.2^b^^,^^d^
BL	3.12 ± 0.37^a^	94.43 ± 17.39^a^^,^^d^	0.94 ± 0.04^a^^,^^c^	145 ± 10^a^	3.9 ± 2.3^a^^,^^c^^,^^d^
DEC	3.15 ± 0.35^a^	99.35 ± 14.81	0.96 ± 0.04^b^	148 ± 11^a^	4.8 ± 2.2^b^
HON	3.20 ± 0.37	101.27 ± 18.08^b^	0.97 ± 0.04	149 ± 11^a^	4.8 ± 2.5^a^^,^^b^
**Run**					
BL	3.59 ± 0.47	115.31 ± 24.94	0.92 ± 0.04	163 ± 10	6.4 ± 2.6
DEC	3.64 ± 0.50	118.68 ± 26.54	0.93 ± 0.03	162 ± 10	6.8 ± 3.0
HON	3.56 ± 0.46	115.73 ± 25.29	0.93 ± 0.03	162 ± 9	6.0 ± 2.5

*NB, Significantly different from; TT*,

a *p < 0.05; BL*,

b *p < 0.05; DEC*,

c *p < 0.05; HON*,

d *p < 0.05*.

Despite these observations, mean HR and $\dot{\text{V}}$O_2_ values did not significantly differ between BL, HON, and DEC cycling (*p* > 0.05). Although no significant physiological differences were evident between HON and DEC cycling, it is noteworthy that only HON had a mean $\dot{\text{V}}$O_2_ which was not significantly lower than TT (*p* > 0.05). Mean intensity during each triathlon run corresponded to 92, 91, and 92% of HR_peak_, and 87, 86, and 88% of $\dot{\text{V}}$O_2peak_, for BL, HON, and DEC, respectively. As summarized in Table 2, there were no significant differences in mean physiological responses during BL, HON, and DEC running (*p* > 0.05).

Magnitude-based inferences suggested that the likelihood of a practically meaningful elevation in all physiological responses during TT vs. the cycling section of all triathlon trials ranged from *likely* to *almost certain* (i.e., 82 to 100% likelihood of being meaningfully higher during TT). Likewise, almost all physiological responses were *possibly* to *almost certainly* higher during DEC and HON cycling compared to BL (i.e., 62 to 98% likelihood of being meaningfully higher vs. BL), with mean $\dot{\text{V}}$O_2_ the only exception. As such, it was *likely* (i.e., 90% certain) that any difference in mean $\dot{\text{V}}$O_2_ between DEC and BL cycling sections was trivial. Mean physiological responses during DEC and HON cycling were of trivial or unclear difference. During running, most of the practically meaningful physiological differences were seen between HON and DEC, with $\dot{\text{V}}$O_2_, $\dot{\text{V}}$_E_, and [BLa^−^] values being either *likely* or *possibly* lower during HON (i.e., 58 to 81% likelihood of a meaningful difference).

**Figure 2:**
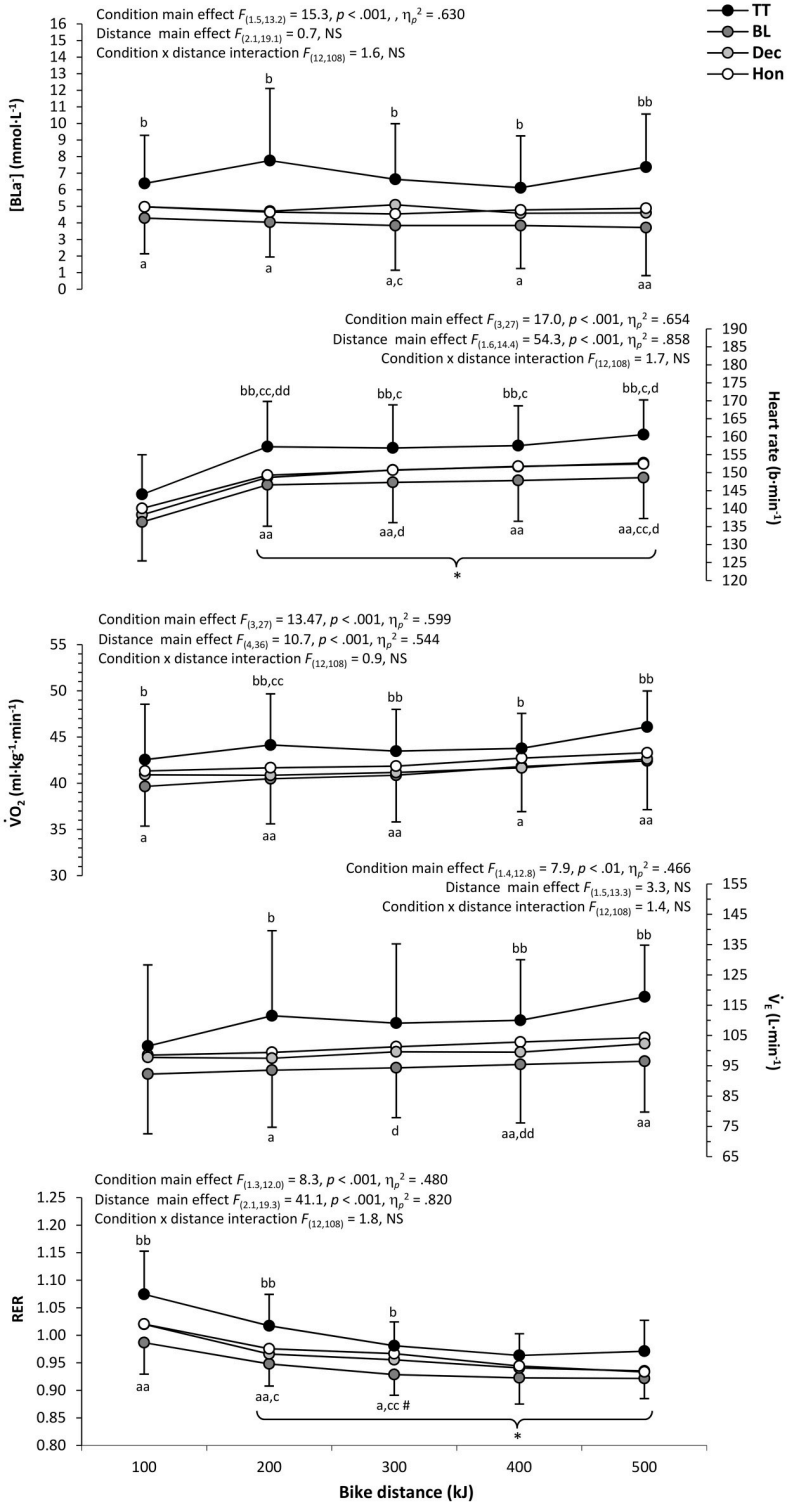
**Mean ± ***SD*** physiological responses for each 100 kJ cycling section**. Significantly different from; TT, ^a^*p* < 0.05, ^aa^*p* < 0.01; BL, ^b^*p* < 0.05, ^bb^*p* < 0.01; DEC, ^c^*p* < 0.05, ^cc^*p* < 0.01; HON, ^d^*p* < 0.05, ^dd^*p* < 0.01; initial value, ^*^p < 0.05; previous value, ^#^*p* < 0.05 (parentheses indicate significance in all conditions).

**Figure 3:**
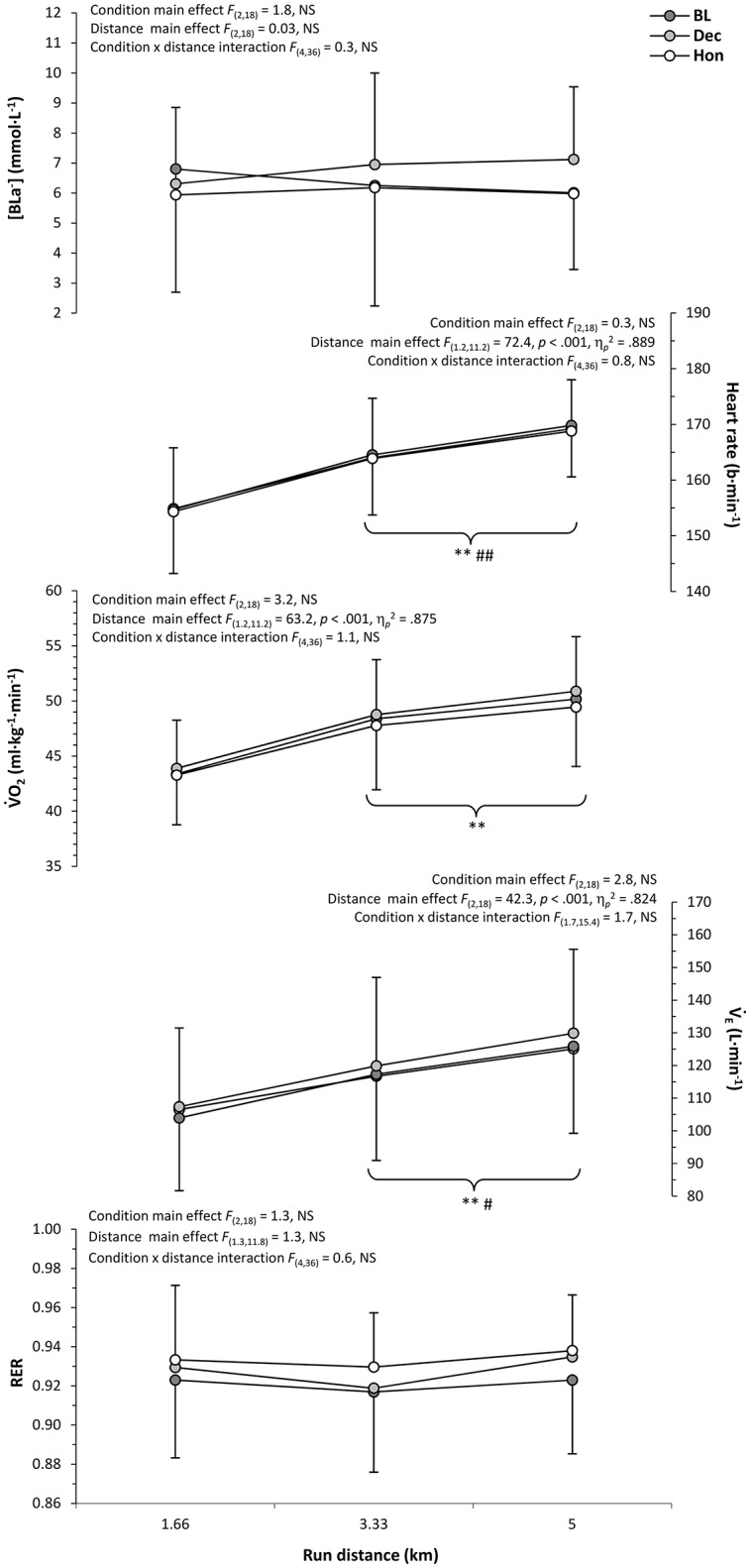
**Mean ± ***SD*** physiological responses for each 1.66 km run section**. Significantly different from; initial value, ^**^p < 0.01; previous value, ^#^*p* < 0.05, ^*##*^*p* < 0.01 (parentheses indicate significance in all conditions).

Figure 2 profiles the physiological responses during simulated triathlon and isolated cycling bouts, including the outcomes of two-way (trial x distance) ANOVA's and *post-hoc* comparisons. As such, significant main effects of cycling condition were found for all physiological measures, whilst there were main effects for distance on HR, $\dot{\text{V}}$O_2_, and $\dot{\text{V}}$_E_ (*p* < 0.05). No significant condition × distance interactions were found for any physiological measure (*p* > 0.05). *Post-hoc* analysis revealed much of the disparity in physiological response to be between BL and TT trials conditions, with direct comparisons of HON and DEC data revealing no significant differences (*p* > 0.05). However, there was a trend for respiratory measures during HON to be higher than DEC, which was indirectly supported by the disparity in significant differences when comparing each of these trials with BL and/or TT. Significant main effects of distance on physiological responses (HR and RER) were found to be a result of significant differences in all conditions between measures taken during the first 100 kJ section and all subsequent measurement intervals. The profile of physiological response during each simulated triathlon run is detailed in Figure 3, which also includes results of primary and *post-hoc* statistical analysis. As suggested by Table 2, there were no significant main effects of prior cycling condition on any physiological measure during running, nor were any significant condition × distance interactions evident (*p* > 0.05). However, significant main effects of run distance were found for HR, $\dot{\text{V}}$O_2_, and $\dot{\text{V}}$_E_ (*p* < 0.05), with all trials demonstrating significant increases in HR and $\dot{\text{V}}$_E_ from each 1.66 km section to the next.

### Perceptual measures

Table 3 summarizes group mean perceptual responses during the completion of TT and triathlon cycling trials. As such, no significant differences in perceptual strain were elicited by the swim section of each triathlon. Furthermore, there were no statistically significant differences between triathlon trials in mean perceptual responses during cycling or running. It was evident that TT cycling was associated with significantly higher mean RPE compared to all bouts of triathlon cycling. It is also noteworthy that only during HON were there no other significant differences in mean perceptual response compared to those during TT.

**Table 3:** **Mean ± ***SD*** perceptual responses during BL, DEC, HON, and TT trials (***n*** = 10)**.

	**Exertion**	**Effort**	**Muscular Pain**	**Thermal Discomfort**	**Breathlessness**	**Arousal**	**Affect**
**SWIM**							
BL	12.5 ± 2.1	12.8 ± 2.1	2.7 ± 1.2	2.3 ± 1.3	3.6 ± 1.4	4.1 ± 1.0	1.4 ± 1.8
DEC	11.8 ± 1.8	12.2 ± 1.7	2.5 ± 1.3	2.3 ± 0.8	3.0 ± 1.0	4.3 ± 1.0	1.4 ± 1.8
HON	11.9 ± 2.2	12.0 ± 2.6	2.9 ± 2.1	2.1 ± 0.9	2.5 ± 1.7	3.7 ± 1.1	2.1 ± 2.1
**CYCLE**							
TT	16.3 ± 1.5^b^^,^^c^^,^^d^	16.3 ± 1.7	6.6 ± 1.9	5.5 ± 1.9	6.5 ± 1.6^b^^,^^c^	4.8 ± 1.0	−1.1 ± 2.0
BL	15.1 ± 1.3^a^	15.4 ± 1.6	5.5 ± 1.4	4.0 ± 1.2	5.2 ± 1.3^a^	4.8 ± 1.0	−0.2 ± 1.8
DEC	15.3 ± 1.6^a^	15.4 ± 1.8	5.8 ± 1.9	4.4 ± 1.2	5.0 ± 1.6^a^	4.7 ± 0.9	−0.4 ± 2.1
HON	15.0 ± 1.7^a^	15.5 ± 2.0	5.8 ± 1.7	4.2 ± 1.3	5.2 ± 1.7	4.8 ± 1.1	−0.3 ± 2.2
**RUN**							
BL	16.9 ± 1.5	17.0 ± 1.6	7.3 ± 1.9	6.4 ± 1.5	7.8 ± 1.6	5.4 ± 0.9	−1.4 ± 2.2
DEC	16.5 ± 1.8	16.9 ± 2.3	7.1 ± 1.9	5.8 ± 2.1	7.3 ± 2.0	5.3 ± 0.9	−0.8 ± 2.6
HON	16.6 ± 1.9	16.7 ± 2.2	7.1 ± 2.0	5.8 ± 1.9	7.4 ± 2.0	5.3 ± 1.0	−1.5 ± 2.6

*NB, Significantly different from; TT*,

a *p < 0.05; BL*,

b *p < 0.05; DEC*,

c *p < 0.05; HON*,

d *p < 0.05*.

Based on magnitude-based inferences, mean perceptual response during TT vs. the cycling section of all triathlon trials was *likely* to *almost certainly* higher for all measures (i.e., 71 to 99% likelihood), except for affect and arousal. As such, mean affect was *likely* lower during TT vs. all other bouts of cycling (i.e., 81 to 89% likelihood). In the case of arousal, there were no clearly meaningful differences evident, with the most likely outcome being a *trivial* difference between trials (i.e., 55 to 75% likelihood). Comparisons between BL, DEC, and HON cycling revealed *trivial* or *unclear* differences in almost all perceptual responses, with thermal strain being the only exception to this. As such, thermal strain was *likely* higher during DEC compared to BL (i.e., 88% certain). During running, thermal strain was again one of few perceptual responses to meaningfully differ between trials, being *likely* lower during both HON and DEC (i.e., 92 and 93% certainty, respectively), compared to BL. The only meaningful difference in perceived exertion was a *possibly* lower mean score during DEC vs. HON running (i.e., 67% certain). Further to this, differences in affect were limited to DEC being *likely* higher (i.e., more positive) than both BL and HON (i.e., 84 and 82% certainty, respectively).

Based on magnitude-based inferences, a meaningfully higher mean perceptual response during TT vs. the cycling section of all triathlon trials ranged from *likely* to *almost certain* for all measures (i.e., 71 to 99% likelihood), except for affect and arousal. As such, mean affect was *likely* lower during TT vs. all other bouts of cycling (i.e., 81 to 89% likelihood). In the case of arousal, there were no clear or meaningful differences evident, with the most likely outcome being a *trivial* difference between trials (i.e., 55 to 75% likelihood). Comparisons between BL, DEC, and HON cycling sections revealed *trivial* or *unclear* differences in almost all mean perceptual responses, with thermal strain being the only exception to this. As such, mean thermal strain was *likely* higher during DEC compared to BL (i.e., 88% certain). During running, thermal strain was again one of few perceptual responses to meaningfully differ between trials, being *likely* lower during both HON and DEC (i.e., 92 and 93% certainty, respectively), compared to BL. The only meaningful difference in perceived exertion was a *possibly* lower mean score during DEC vs. HON running (i.e., 67% certain). Further to this, differences in affect were limited to DEC being *likely* higher (i.e., more positive) than both BL and HON (i.e., 84 and 82% certainty, respectively).

**Figure 4:**
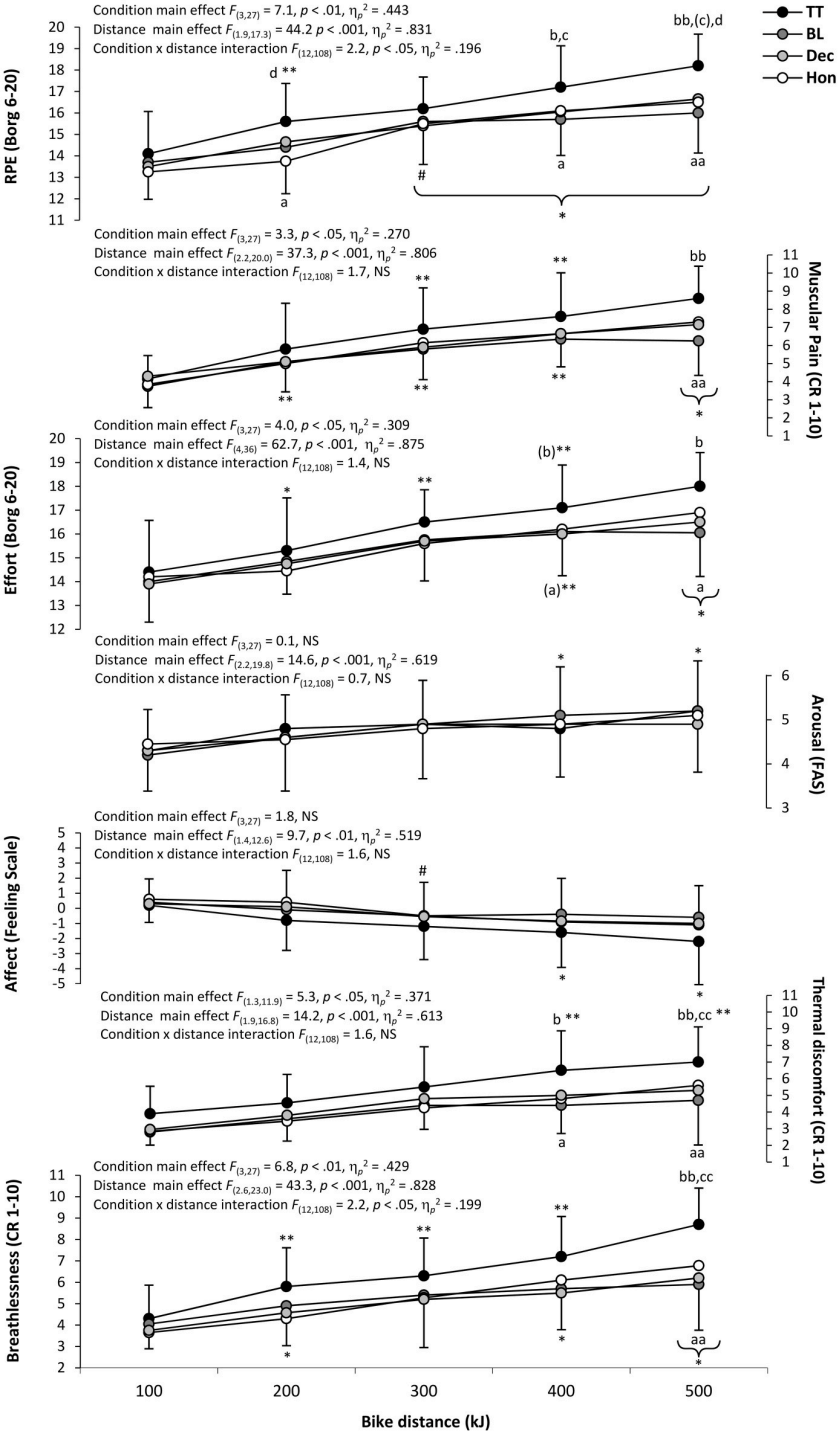
**Mean ± ***SD*** perceptual responses for each 100 kJ cycle section**. Significantly different from; TT, ^(*a*)^*p* = 0.051, ^a^*p* < 0.05, ^aa^*p* < 0.01; BL, ^(*b*)^*p* = 0.051, ^b^*p* < 0.05, ^bb^*p* < 0.01; DEC,^(*c*)^*p* = 0.051, ^c^*p* < 0.05, ^cc^*p* < 0.01; HON, ^d^*p* < 0.05, ^dd^*p* < 0.01; initial value, ^*^p < 0.05, ^**^p < 0.01; previous value, ^#^*p* < 0.05 (parentheses indicate significance in all conditions).

**Figure 5:**
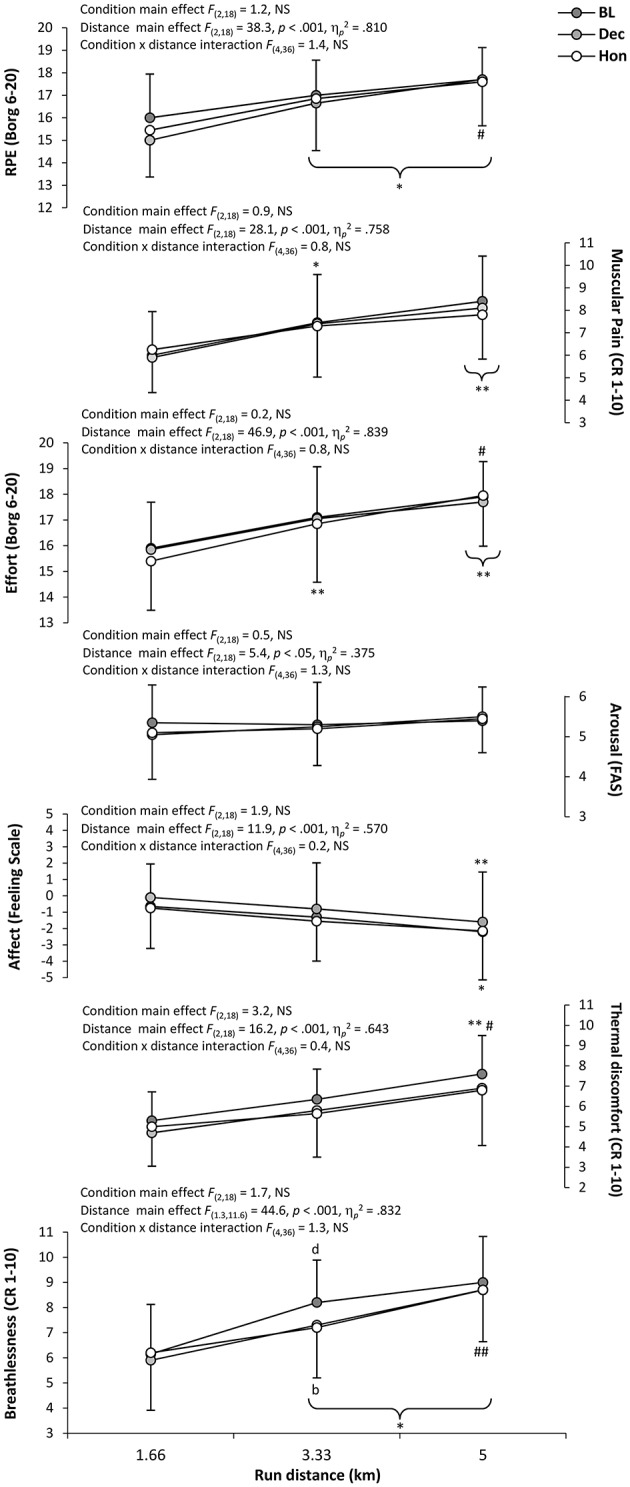
**Mean ± ***SD*** perceptual responses for each 1.66 km run section**. Significantly different from; BL, ^b^*p* < 0.05; HON, ^d^*p* < 0.05; initial value, ^*^p < 0.05, ^**^p < 0.01; previous value, ^#^*p* < 0.05, ^*##*^*p* < 0.01 (parentheses indicate significance in all conditions).

Distance profiles (and associated statistical outcomes) of perceptual measures during cycling and running sections of each trial are presented in Figures 4, 5, respectively. Significant distance effects were found for all perceptual measures during cycling (*p* < 0.05), whilst a significant main condition effect was evident for all perceptual responses except for affect and arousal (*p* > 0.05). A significant condition × distance interaction was only apparent for RPE and breathlessness (*p* < 0.05). During running, significant distance effects were found for all perceptual measures (*p* < 0.05), although no condition effects or condition × distance interactions were evident for any perceptual response (*p* > 0.05). Further to these findings, collated individual perceptual responses across the duration of each triathlon trial revealed strong correlations with the percentage of overall triathlon time completed (*r* = 0.92–0.97, *p* < 0.05). Repeated-measures ANOVA showed the relationship (i.e., *r* coefficient) between individual participants' perceptual status and percentage of overall triathlon time was largely unaffected by cycling condition, with no statistically significant main effects found (*p* > 0.05).

**Figure 6:**
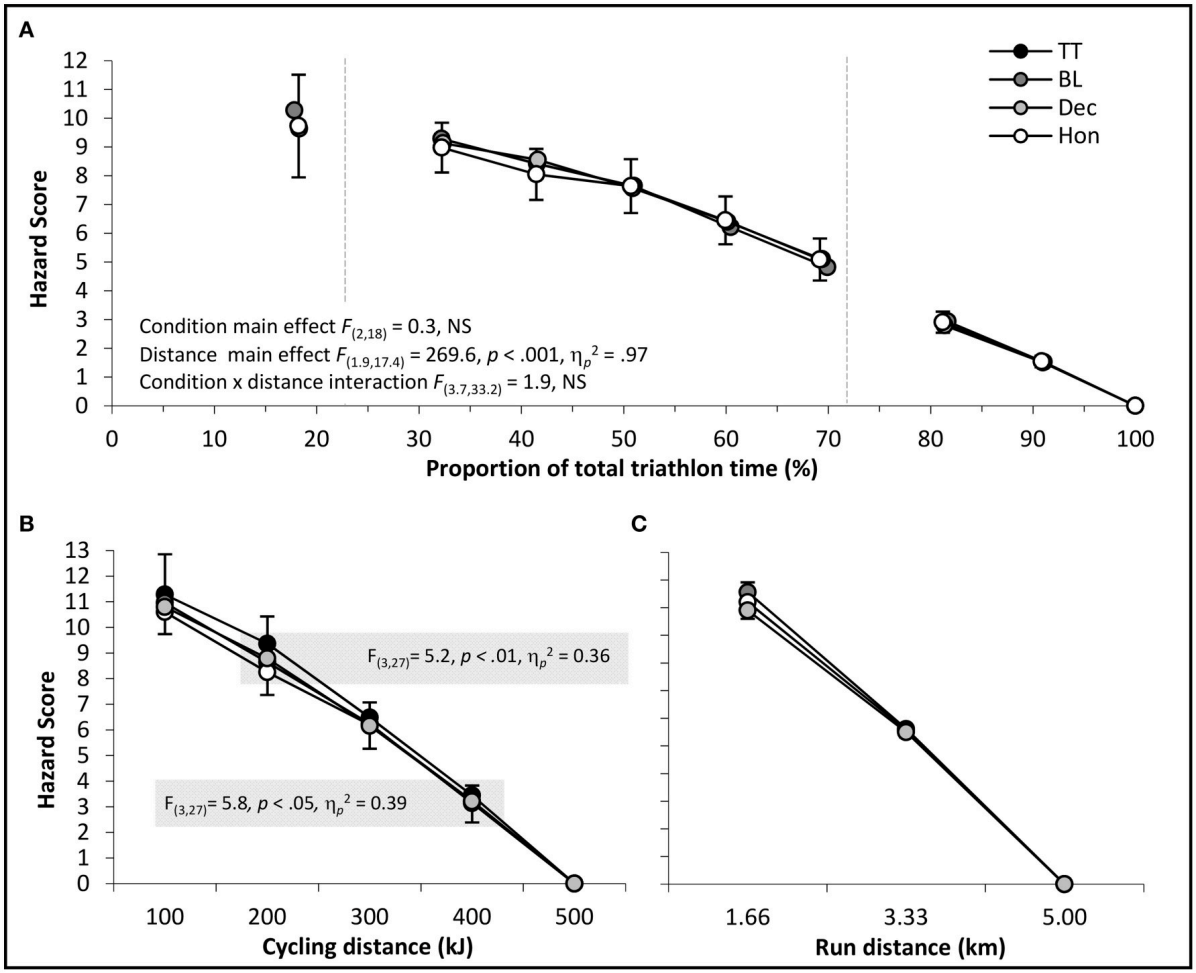
**Mean ± ***SD*** Hazard Scores in relation to (A)** the proportion of total triathlon distance remaining, **(B)** the proportion of the bike section remaining, **(C)** the proportion of run distance remaining (dashed lines indicate transition end).

As a simple index of the momentary risk perception associated with pacing behavior, the so-called “Hazard Score” (de Koning et al., 2011) was individually calculated and profiled across each triathlon trial by multiplying RPE values by the proportion of overall triathlon distance remaining at that particular point in time (Figure 6A). Analysis via two-way repeated-measures ANOVA failed to show a significant main effect on Hazard Score for triathlon condition or a significant condition × distance interaction, although there was a significant main effect for total triathlon distance. Hazard Scores were also calculated specifically for cycling and running sections by multiplying reported RPE values by the proportion of discipline-specific distance remaining at that point. For cycling-specific Hazard Scores (Figure 6B), two-way repeated-measures ANOVA showed significant main effects for condition [*F*_(3.0, 27.0)_ = 4.5, *p* < 0.05, η_*p*_^2^ = 0.33] and distance [*F*_(1.5, 13.6)_ = 1029.1, *p* < 0.001, η_*p*_^2^ = 0.99], although no significant condition-by-distance interaction was seen (*p* > 0.05). *Post-hoc* analysis highlighted that between-condition differences during cycling were attributable to the Hazard Scores of TT, which were significantly higher compared to HON at 200 kJ (*p* < 0.05), and vs. both BL and DEC at 400 kJ (*p* < 0.05). The same analysis of running-specific Hazard Scores (Figure 6C) failed to show a significant main condition effect or significant condition x distance interaction (*p* > 0.05), although there was a significant main effect for running distance [*F*_(1.1, 10.0)_ = 684.2, *p* < 0.001, η_*p*_^2^ = 0.99).

## Discussion

The aim of this study was to ascertain the effects of deceptively aggressive bike pacing on performance, and associated physiological and perceptual responses, during simulated sprint-distance triathlon. With this in mind, the experimental hypothesis that cycling closer to the highest sustainable intensity (i.e., mean isolated time trial power output) would improve previous best simulated triathlon performance was accepted. This was the case irrespective of whether or not triathletes were made aware of this relatively aggressive pacing strategy. The decision to accept this hypothesis was based on the finding of significant (*p* < 0.05) and almost certainly meaningful improvements in the overall simulated triathlon times of both HON and DEC, compared to that of previous best (i.e., BL) performance. Similarly, the hypothesis that making triathletes aware of aggressive cycle pacing would impair subsequent run and overall performance, relative to that of a deceptive pacing condition, was also accepted. This decision was made in light of the significant (*p* < 0.05) and probably meaningful improvements in running time during DEC, compared to BL, and the apparent failure of triathletes to significantly or meaningfully improve on their BL run performance during HON (*p* > 0.05, possibly trivial/unclear difference). Furthermore, whilst the 17 s difference between HON and DEC running times did not reach statistical significance, it would appear *probable* or *likely* that this represents a meaningfully quicker run performance during DEC. Indeed, the differences in running performance between DEC and the relatively slower BL and HON trials are comparable to those observed during the deceptively manipulated triathlon running trials of Taylor and Smith (2014). As highlighted by these authors, such differences cannot be ignored given that an average of only 9 s can separate the run and overall event ranking positions for of the top 20 sprint-distance triathletes at (age-group) World Championship level (ITU, 2012).

The current study findings therefore extend those of previous deception research to offer further evidence that expectations and beliefs regarding a particular exercise task and/or intervention are likely to influence athletes' perception of internal and external stimuli, and the subsequent conscious (anticipatory) pacing decisions they make in attempting to optimize performance (Micklewright et al., 2010; Stone et al., 2012; Taylor and Smith, 2014; Williams et al., 2014, 2015; Waldron et al., 2015; Shei et al., 2016). It has been speculated that this is the case during multi-modal exercise (Hausswirth et al., 1999), with previous simulated triathlon studies finding that a relatively aggressive mid-event (i.e., cycling) pacing strategy leads to subsequent reductions in running performance (Hausswirth et al., 1999; Suriano and Bishop, 2010). However, this is the first study to offer clear experimental evidence in support of this suggestion, with the superior running performance of DEC illustrating that expectations regarding aggressive mid-event pacing can strongly influence subsequent exercise intensity regulation and performance during multi-modal exercise. As such, the profile of run pacing during DEC revealed a more aggressive starting strategy coupled with earlier initiation of an end-spurt, relative to BL and HON trials (Figure 1B).

It would therefore appear that deceptively aggressive bike pacing allows triathletes to maximize their sustainable intensity in this discipline, without the subsequent impairments in running performance which are typically seen when athletes are made aware of this mid-event cycling strategy. This corroborates with the suggestion that athletes perceive higher and/or earlier than anticipated levels of exercise intensity as posing a greater risk to the completion of an exercise task and, therefore, as having a “price to pay” at a later stage of performance (i.e., reduction in running pace to maintain sufficient reserve and avoid premature exhaustion or risk of harm) (Cohen et al., 2013; Micklewright et al., 2015). Task-specific expectations and beliefs therefore appear to play a key role in determining how much reserve capacity individuals are willing and able to utilize in the pursuit of optimal self-paced multi-modal exercise performance. With this in mind, there may be a common need, particularly amongst non-elite sprint-distance triathletes, to “relearn” what constitutes an optimal pacing strategy across the entire event. More specifically, if triathletes are to optimize short-distance event performance then it would appear that the holding back of any reserve capacity should be minimized during the cycle section. That is, the highest sustainable intensity should be maintained so as to replicate isolated time-trial performance as closely as possible, as suggested by Suriano and Bishop (2010). Likewise, the highest sustainable (even) pace should be established during the early stages of the triathlon run so that there is minimal available reserve with which to perform a final end-spurt. However, given that the pacing template of experienced triathletes is likely to be well-established (Baron et al., 2011) further research is needed to establish the extent to which such “re-education” of pacing is possible, how it may be facilitated by sports scientists and coaches, and ways in which such deviation from a previously-favored pacing strategy may be influenced by individual risk-perception and risk-taking traits (Micklewright et al., 2015).

As highlighted in a recent review of factors influencing pacing during triathlon (Wu et al., 2014), it is evident that the perceptual mechanisms underpinning multi-modal endurance performance have been largely neglected by research to date. Indeed, whilst a number of studies have examined the physiological responses of triathletes to manipulations of cycling intensity (Hausswirth et al., 1999, 2001; Solano et al., 2003; Suriano and Bishop, 2010), this is the first study to have considered how a number of perceptual responses may also be influenced by the relative intensity of triathlon-specific cycling and subsequent running. Furthermore, the diversity and frequency of physiological measures obtained during the current study offers a previously unavailable profile of how these responses may develop as a result of both deceptive and non-deceptive manipulations of cycle pacing during complete triathlon performance. Generally, it would appear that levels of physiological and perceptual strain increased with higher cycling intensities during the current study, with little, if any, substantial difference in physiological and perceptual response during each triathlon run. There was also a broad trend for physiological and perceptual strain to increase as a greater proportion of each discipline, and overall triathlon performance, was completed (Figures 2–5).

These observations underline the suggested “holding back” of a progressively decreasing reserve capacity over the course of “fastest possible” triathlon performance (i.e., “BL”). They would also appear to confirm that the anticipatory process of reserve maintenance is sensitive to levels of both physiological and perceptual strain during self-paced multi-modal exercise (Swart et al., 2009; Tucker, 2009). However, it is evident that any differences in physiological or perceptual response observed during each simulated triathlon trial were much more subtle than those seen for performance-related measures, particularly when comparing HON and DEC trials. The failure to establish clear links between physical and/or perceptual responses and performance is not uncommon in contemporary pacing research (Micklewright et al., 2010; Jones et al., 2014; Rhoden et al., 2014). Indeed, such findings reinforce the view that psychophysiological processes interact in a complex and multidimensional manner during the regulation of self-paced exercise performance (Renfree et al., 2012; Jones et al., 2014). As such, the methods used to examine physical and perceptual factors during future studies may need further refinement (e.g., increased frequency, consideration of the specific thoughts of participants) to be able to more clearly understand their interaction and influence during self-paced multi-modal exercise.

With this in mind, the authors are cognizant of the fact that there are potential limitations within the current study design which may have impacted the strength with which it was able address the key aims and hypotheses. Indeed, it could be argued that the counterbalancing of HON and DEC trials may have led to some participants becoming more, or less, consciously attuned to the demands of aggressive cycle pacing by the time they were exposed to DEC. Although post-experimental debriefs suggested that this was not the case, such an ordering effect could have made it less likely for those completing HON first to have been truly deceived about their pacing during their subsequent DEC performance. At the very least, the different ordering of DEC and HON trials may have the potential to influence the perceptual responses of participants and so should be considered as a limitation of the current study. Indeed, whilst participants did not report being consciously aware of any deceptive manipulation, it was evident from a number of debrief interviews that their prior experiences of either DEC and HON somehow served to “frame” their approach to, and interpretation of, subsequent performance trials. Whilst this view corroborates with previous work focusing on the effects of prior experiences during relatively short single-mode endurance performance (e.g., Micklewright et al., 2010), it is certainly a line of study which would be of value for researchers to explore during multi-modal endurance performance. That said, the value of randomization and counterbalancing of experimental conditions within a repeated-measures study design cannot be ignored, given that not doing so may clearly be criticized for introducing confounding ordering or time-related effects (i.e., learning/familiarity, fatigue, training/fitness status, equipment). Whilst the authors are therefore confident in the robustness of the current study design, such findings must always be viewed with a degree of caution in light of the specific context of the study and the possible limitations associated with the particular approach taken.

Irrespective of these points, the current study provides valuable and novel evidence with which to address some the ongoing challenges to RPE being considered as the chief perceptual mediator of pace regulation during exercise. Indeed, based on their observations during and after aggressive mid-event pacing during single-mode (cycling) exercise, Cohen et al. (2013) concluded that RPE may be less closely tied with deviations away from template power output (i.e., reserve access) than is proposed by the “anticipatory-RPE” model of Tucker (2009). The current study would appear to lend some support to this suggestion during multi-modal exercise, given the lack of any significant difference in RPE during each simulated triathlon. Furthermore, the conversion of RPE values into a supposedly more meaningful index of pacing “riskiness” (i.e., the Hazard Score of de Koning et al., 2011) failed to distinguish between each triathlon trial of the present study, despite substantial differences in pacing and performance between cycling and running sections of each trial. On the other hand, some of the current study observations would still seem to suggest that triathletes utilize discipline-specific templates to interpret and manage levels of psychophysiological strain, and that these templates can be influenced by task-specific beliefs and expectations. Indeed, whilst they were not statistically different, if the profiles of RPE increase during each period of triathlon cycling were maintained beyond the end of the discipline (i.e., projected forward), then an RPE value of 20 (i.e., “maximal exertion”) would not have been reached until 130, 108, and 103% of the total triathlon duration for BL, DEC, and HON, respectively. Extending the findings of Taylor and Smith (2014), this would appear to further illustrate the supposed role of RPE in maintaining a reserve capacity during “fastest possible” self-paced triathlon performance (i.e., BL trial) and highlight the subtle, but practically meaningful, effects of deception on the regulation and forecasting of RPE during individual triathlon modalities, both of which are indicative of discipline-specific RPE templates. However, it is important to note that these between trial differences in projected levels of psychophysiological strain were not exclusive to RPE and were evident in the profiles of all other perceptual responses.

Given these points, it is appears likely that an array of psychophysiological factors may indeed influence pacing decisions during exercise, possibly by way of “fine-tuning” the “coarse” relationship between RPE growth and momentary power output (Cohen et al., 2013). This suggestion is not unique, with a growing number of contemporary pacing studies theorizing that perceptions other than RPE (e.g., sense of effort, perceived muscular pain, breathlessness, thermal strain, and affect) are of equal, if not greater, importance to anticipatory pace regulation and reserve capacity maintenance (Micklewright et al., 2010; Renfree et al., 2012; Stone et al., 2012; Jones et al., 2014; Pageaux, 2014; Williams et al., 2014, 2015). In particular, an individual's affective status has been suggested as a potentially more influential mediator of pace regulation than RPE (Baron et al., 2011; Jones et al., 2014; Renfree et al., 2014). On one hand, it would appear that the findings of the current study fail to support to this suggestion during multi-modal exercise, given the lack of statistically significant difference in affective response during each simulated triathlon. However, there was a *likely* meaningful trend for more positive levels of affect to be sustained throughout the quicker, more aggressive, and thus most physiologically demanding triathlon run, which followed the deceptively aggressive cycling condition. This would corroborate with the view that more negative affect is associated with reduced tolerance of physiological strain and poorer performance (Renfree et al., 2012), although it would also appear to disagree with the findings of Taylor and Smith (2014) which demonstrated more negative levels of affect throughout deceptively quicker, more aggressive, and thus more physiologically stressful, triathlon running. As such, it would seem that performance enhancement by deception may somehow be linked to an altered association between affective status and physiological strain, leading to a greater willingness to persevere with workloads that would otherwise be considered unsustainable.

However, given the difficulty in clearly distinguishing between the affective responses of each triathlon trial of the present study, it is evident that further research is required to confirm and better understand if, how, and why, someone's emotional status (i.e., levels of affect and arousal) may influence pace regulation more than “what” they are feeling (i.e., RPE, effort, thermal discomfort, breathlessness), particularly during multi-modal exercise. With this in mind, it may also be of value for researchers to examine whether the deceptive enhancement of both single and multi-modal performance reflects a change in the specific thoughts of participants, rather than an altered interpretation of common psychophysiological scales (Brick et al., 2016).

## Conclusions

This study has shown that the imposition of deceptively aggressive cycle pacing, derived from previous “fastest possible” self-paced performance, enhances subsequent run and overall performance during simulated sprint-distance triathlon. It also suggests that interceptive sensations associated with fatigue and effort may be perceived differently according to an individual's expectations and beliefs regarding the past, present and future demands of pacing during multi-modal exercise. This would appear to be the case regardless of whether psychophysiological strain is established using RPE or by more distinct measures of interceptive sensations and emotions (i.e., sense of effort, perceived muscular pain, breathlessness, thermal strain, affect, and arousal). Whilst some form of anticipatory “template” may therefore be used by athletes to regulate the development of psychophysiological strain across a particular multi-modal exercise task, it would appear that the influence of afferent feedback on this process can be manipulated to modify pacing and enhance performance. Although these points echo previous conclusions (e.g., Taylor and Smith, 2014) this study demonstrates, for the first time, that the influence of manipulated task beliefs on the interaction between psychophysiological status and pacing can persist across consecutive modes of self-paced exercise, so as to optimize multi-modal performance. As such, it is hoped that the findings of the current study serve to catalyze the exploration and improved understanding of the anticipatory psychophysiological mechanisms which govern pace regulation across consecutive modes of exercise.

## Author contributions

Both of the listed authors made a significant contribution to this study, including conceiving and designing the experiments (DT and MS), collecting, analyzing, and/or interpreting the data (DT and MS), conceptualizing and drafting the initial manuscript (DT), and critically reviewing/revising the manuscript (DT and MS). Further to these points, DT conducted the final approval of the version to be published (in agreement with MS) and is accountable for all aspects of the work in ensuring that questions related to the accuracy or integrity of any part of the work are appropriately investigated and resolved.

### Conflict of interest statement

The authors declare that the research was conducted in the absence of any commercial or financial relationships that could be construed as a potential conflict of interest.
